# Worldwide Susceptibility Rates of *Neisseria gonorrhoeae* Isolates to Cefixime and Cefpodoxime: A Systematic Review and Meta-Analysis

**DOI:** 10.1371/journal.pone.0087849

**Published:** 2014-01-31

**Authors:** Rui-xing Yu, Yueping Yin, Guan-qun Wang, Shao-chun Chen, Bing-jie Zheng, Xiu-qin Dai, Yan Han, Qi Li, Guo-yi Zhang, Xiangsheng Chen

**Affiliations:** 1 Reference STD Lab, National Center for STD Control, Chinese CDC, Institute of Dermatology, Chinese Academy of Medical Sciences, Peking Union Medical College, Jiangsu Key Laboratory of Molecular Biology for Skin Diseases and STIs, Nanjing, Jiangsu Province, China; 2 Department of STD, Anhui Provincial Institute of Dermatology, Hefei, Anhui Province, China; California Department of Public Health, United States of America

## Abstract

**Background:**

*Neisseria gonorrhoeae* (NG) infection is a serious public health problem. The third-generation extended-spectrum cephalosporins (ESCs) have been used as the first-line treatment for NG infection for almost three decades. However, in recent years, treatment failures with the oral third-generation ESCs have been reported worldwide. This study aimed to estimate worldwide susceptibility rates of NG to cefixime and cefpodoxime by analyzing data from all relevant published studies.

**Methodology/principal findings:**

Two researchers independently searched five databases to identify studies on susceptibilities of NG to cefixime and cefpodoxime published between January 1, 1984 and October 15, 2012. A fixed-effect model was used to perform group analysis, and a χ2 test was employed to make subgroup comparison. Publication bias was assessed with the Begg rank correlation test. The pooled susceptibility rate of NG isolates to cefixime was 99.8% (95% CI: 99.7%–99.8%). The cefixime susceptibility rate of NG isolates from men was significantly lower than that from patients without information of gender or from men and women; the susceptibility rate of NG isolates from Asia was significantly lower than that from other continents; and the susceptibility rate of NG isolates collected before or during 2003 was significantly higher than that after 2003. The pooled susceptibility rate of NG isolates to cefpodoxime was 92.8% (95% CI: 89.0%–95.3%), which was lower than that to cefixime (92.8% vs. 99.8%, χ2 = 951.809, P<0.01).

**Conclusions:**

The susceptibility rate of NG isolates to cefixime varied with the gender of patients and geographical location from which NG isolates were collected, and declined with time. The reported lower susceptibility rate of NG isolates to cefixime and associated treatment failures, as well as the emergence of NG strains with cephalosporin resistance call for the more effective control of NG infection and the development of new antibiotics.

## Introduction

*Neisseria gonorrhoeae* (NG) is a common sexually transmitted pathogen which causes male urethritis and female endocervicitis. The World Health Organization (WHO) estimated that there were 106 million new cases of gonorrhea worldwide in 2008 [1]. During the past three decades, NG has developed resistance to most of the antibiotics used to treat gonorrhea, including penicillin, tetracyclines, and fluoroquinolones [2], [3], [4]. In the 1990s, the third-generation extended-spectrum cephalosporins (ESCs) were recommended internationally to treat NG infection [5]. However, during the past two decades, there have been reports on verified treatment failures with cefixime in Japan, France, Norway, Austria, United Kingdom, and Canada; with ceftriaxone in Sweden and Australia; with ceftibuten in Hong Kong; and with cefdinir in Japan [6], [7], [8], [9], [10], [11], [12], [13], [14], [15], [16]. As a result, the oral ESCs are not currently recommended for treating NG infection in Japan, urban areas of Australia, Europe, and United States [17], [18], [19], [20]. However, cefixime is still the recommended drug for NG infection in Canada [21]. According to the Clinical and Laboratory Standards Institute (CLSI), the minimum inhibitory concentration (MIC) breakpoint for oral ESCs cefixime and cefpodoxime susceptibility were <0.25 mg/L and 0.5 mg/L respectively [22]. To date, there have been many studies on antimicrobial susceptibilities of NG to cefixime and cefpodoxime. The aim of this study were: (1) to estimate the susceptibility rates of NG to cefixime and cefpodoxime worldwide from relevant studies; (2) to compare cefixime susceptibility rate of NG isolates collected from different populations and locations, and its development over time.

## Methods

### Literature search

Two independent researchers (RY and GW) searched five databases (PubMed, Embase, Web of Science, CNKI, and Wanfang) to identify relevant studies published from August 1984 to October 2012. Search terms included “*Neisseria gonorrhoeae*,” “*gonorrhea*,” or “*gonococcus*”; and their combinations with “*cefixime*” or “*cefpodoxime*,” and with subject headings “*MIC*,” “*minimum inhibitory concentration*,” “*resistance*,” “*resistant*,” “*susceptible*,” or “*susceptibility*.” (See Table S1) References cited in the retrieved articles were also screened, and duplicated reports were excluded. This review was conducted in four stages (identification, screening, eligibility assessment, and inclusion) according to the Preferred Reporting Items for Systematic Reviews and Meta-Analyses (PRISMA) guidelines [23], [24]. (See Table S2)

### Eligibility criteria and validity assessment

The included studies met the following criteria: (1) original studies published between January 1, 1984 and October 15, 2012 in any language; (2) specified the total number of NG isolates; (3) determined the MICs of cefixime or cefpodoxime with agar dilution method; and (4) following CLSI standards, reported the antimicrobial susceptibility rate in NG isolates, or implied it by indicating their MICs of cefixime or cefpodoxime, and/or the number of non-susceptible NG isolates. According to the above criteria, the eligibility and validity of selected studies were assessed independently by two researchers (RY and GW), any disagreement was resolved by involving the third researcher (YY).

### Data extraction

Data was extracted from each included study and compiled under the following categories using a standardized form: (1) first author and publication year; (2) location (country and city) where the study was conducted; (3) isolates collection period; (4) study population, if available; (5) drugs: cefixime and/or cefpodoxime; (6) number of tested isolates; and (7) susceptibility rate (**Table 1**). The data was extracted independently by two authors (RY and GW), any resultant discrepancies were resolved by involving the third researcher (YY).

**Table 1:** Overview of 25 included studies on cefixime and cefpodoxime susceptibility rates of NG isolates.

Study number	First author, year	Location	Isolate collection period^a^	Population^b^	Drug	No. isolates	Susceptibility rate(%)^c^
1	Fekete T, 1991 [50]	Philadelphia and San Diego, USA	-	Mix	Cefpodoxime	77	100
2	Kohl PK, 1995 [41]	Heidelberg, Germany	1986–1990	Mix	Cefixime	203	100
3	Lewis DA, 1995 [42]	East London, England	-	Patients	Cefixime	104	100
4	Tapsall JW, 1995 [51]	Sydney, Australia	09/1993–12/1993	Mix	Cefpodoxime	137	100
5	Lewis DA, 1996 [34]	London, England	04/1992–03/1993	Patients	Cefixime	378	100
6	Nissinen A, 1997 [35]	Finland	1993	Mix	Cefixime	337	98
7	Fox KK, 1997 [39]	Surveillance sites, USA	1992 and 1994	Patients	Cefixime	10402	99.856
			1992	Patients	Cefixime	5406	99.8
			1994	Patients	Cefixime	4996	99.92
8	Komeda H, 2004 [47]	Ogaki, Japan	1998–2002	Men	Cefpodoxime	147	88.8
			1998	Men	Cefpodoxime	28	93.3
			1999	Men	Cefpodoxime	30	96.8
			2000	Men	Cefpodoxime	30	88.2
			2001	Men	Cefpodoxime	30	82.9
			2002	Men	Cefpodoxime	29	89.1
9	Shigemura K, 2004 [44]	Hyogo and Osaka, Japan	2004	Men	Cefixime	87	100
10	Kagami Y, 2005 [40]	Tokyo, Japan	1999–2004	Men	Cefixime	281	95.7
			1999	Men	Cefixime	41	100
			2000	Men	Cefixime	57	93
			2001	Men	Cefixime	24	100
			2003	Men	Cefixime	58	96.6
			2004	Men	Cefixime	101	94.1
11	Donegan EA, 2006 [27]	Bali, Indonesia	08/2004–11/2005	FSWs	Cefixime	147	100
				FSWs	Cefpodoxime		100
12	Zarakolu P, 2006 [46]	Turkey, Ankara	-	Sex workers	Cefixime	30	100
13	De Jongh, 2007 [30]	Pretoria	03/2004–04/2005	Men	Cefpodoxime	141	100
14	Wang S, 2007 [35]	USA	1992–2003	Mix	Cefixime	62461	99.928
15	Palmer HM, 2008 [48]	Scottish	04/2004–03/2006	Mix	Cefixime	1765	100
16	Apalata T, 2009 [38]	Maputo, Mozambique	03/2005–04/2005	Patients	Cefixime	55	100
17	Allen VG, 2011 [31]	Ontario, Canada	10/2008–11/2008	Patients	Cefixime	149	100
18	Endo K, 2011 [28]	Tokyo, Japan	2006–2010	Men	Cefixime	156	86.5
			2006	Men	Cefixime	47	100
			2007	Men	Cefixime	23	100
			2008	Men	Cefixime	18	100
			2009	Men	Cefixime	38	47.4 (outlier)
			2010	Men	Cefixime	30	96.7
19	Lee H, 2011 [33]	Korea	2001–2006	Mix	Cefixime	162	99.38
			2001	Mix	Cefixime	41	100
			2002	Mix	Cefixime	25	100
			2003	Mix	Cefixime	24	100
			2004	Mix	Cefixime	20	95.8
			2005	Mix	Cefixime	21	100
			2006	Mix	Cefixime	31	100
20	Martin I, 2011 [43]	Canada	2000–2009	Mix	Cefixime	10993	99.45
20	Martin I, 2011 [43]		2000	Mix	Cefixime	1206	100
			2001	Mix	Cefixime	1234	100
			2002	Mix	Cefixime	1163	100
			2003	Mix	Cefixime	800	100
			2004	Mix	Cefixime	855	100
			2005	Mix	Cefixime	905	100
			2006	Mix	Cefixime	1532	100
			2007	Mix	Cefixime	1438	99.93
			2008	Mix	Cefixime	947	99.894
			2009	Mix	Cefixime	913	99.56
21	Tanaka M, 2011 [49]	Western, Mid-eastern, Eastern Japan	02/2008–12/2009	Mix	Cefixime	494	99.6
22	Tanaka M, 2011 [29]	Fukuoka, Japan	01/2008–12/2008	Patients	Cefixime	242	98.76
23	Carannante A, 2012 [32]	Italy	2006–2010	Men	Cefixime	293	99.32
24	Mehta S, 2012 [36]	Kisumu, Kenya	2002–2009	Young men	Cefixime	168	100
25	Takahashi S, 2012 [45]	Sapporo, Japan	01/2007–01/2009	Men	Cefixime	51	92.2

a Isolate collection period: “-” means this information unavailable.

b Study population: FSWs (female sex workers); men (men with urethritis); mix (male and female patients), patients (patients whose gender was not identified); young men (young men with discharge); Sex workers (sex workers whose gender was not identified).

c Susceptibility rate = number of susceptible NG isolates/total number of isolates tested×100.

### Quality assessment

A set of criteria including location, isolates collection period, population, isolates identification, NO. of the isolates, NO. of NG isolates ≥100, and control strains these seven factors (Table 2), was used for assessing quality of the included studies. The quality assessment was independently performed by two researchers (RY and GW), and any resultant discrepancies were resolved by involving the third researcher (YY).

**Table 2:** Quality assessment of the studies included in meta-analysis.

Study number	First author, year	Location^a^	Isolates collection period^b^	Population^c^	Isolates identification^d^	NO. isolates^e^	NO. of NG isolates≥100^f^	Control strains^g^
1	Fekete T, 1991	+	−	+	+	+	−	−
2	Kohl PK, 1995	+	+	−	+	+	+	−
3	Lewis DA, 1995	+	−	−	+	+	+	+
4	Tapsall JW, 1995	+	+	−	−	+	+	−
5	Lewis DA, 1996	+	+	−	+	+	+	+
6	Nissinen A, 1997	+	+	+	+	+	+	−
7	Fox KK, 1997	+	+	−	−	+	+	+
8	Komeda H, 2004	+	+	+	−	+	+	−
9	Shigemura K, 2004	+	+	+	−	+	−	+
10	Kagami Y, 2005	+	+	+	−	+	+	−
11	Donegan EA, 2006	+	+	+	+	+	+	+
12	Zarakolu P, 2006	+	−	+	+	+	−	+
13	De Jongh, 2007	+	+	+	+	+	+	+
14	Wang S, 2007	+	+	+	−	+	+	+
15	Palmer HM, 2008	+	+	−	−	+	+	−
16	Apalata T, 2009	+	+	−	+	+	−	+
17	Tanaka M, 2011	+	+	+	+	+	+	+
18	Martin I, 2011	+	+	−	−	+	+	+
19	Lee H, 2011	+	+	+	−	+	+	+
20	Endo K, 2011	+	+	+	+	+	+	+
21	Tanaka M, 2011	+	+	−	−	+	+	−
22	Allen VG, 2011	+	+	−	+	+	+	+
23	Carannante A, 2012	+	+	+	−	+	+	+
24	Takahashi S, 2012	+	−	+	+	+	−	+
25	Mehta S, 2012	+	+	+	+	+	+	−

a “+” means the study specifying the location where NG isolates were collected; “−” stands for this information missing from the study.

b “+” means the study specifying isolates collection period; “−” stands for this information missing from the study.

c “+” means the study describing the population from whom NG isolates were obtained; “−” stands for this information missing from the study.

d “+” means the study describing the method of identifying NG isolates; “−” stands for this information missing from the study.

e “+” means the study indicating the number of tested NG isolates; “−” stands for this information missing from the study.

f “+” means the study including at least 100 tested NG isolates; “−” stands for the study failing to do it.

g “+” means the study utilizing control strains recommended by WHO in determining MICs with agar dilution method; “−” stands for the study failing to do it.

### Statistical analysis

Statistical analyses were performed using Statistical Package for the Social Sciences for Windows (SPSS, version 20.0, Chicago, IL, USA), MetaAnalyst Beta 3.13 software, and Stata 12.0 software. The antimicrobial susceptibility rate in NG isolates with corresponding 95% confidence intervals (CI) was calculated for each individual study. A fixed-effects model was used to perform group analysis. Based on NG isolates collected from different populations and continents in the included studies, a fixed subgroup analysis was used and a χ^2^ test was employed to make subgroup comparison (P<0.05 indicating statistical significance). Furthermore, because the first case of treatment failure with cefixime was reported in 2003 [6], we divided NG isolates into two groups—isolates collected before or during 2003 and those after 2003. Between-study heterogeneity was measured by performing the Q test (P<0.10 indicating statistical significance) and calculating I^2^ values (ranging between 0% and 100%, with lower values representing less heterogeneity) [25]. Publication bias was assessed using the Begg rank correlation test (P<0.05 indicating statistical significance) [26].

## Results

### Study selection

**Figure 1:**
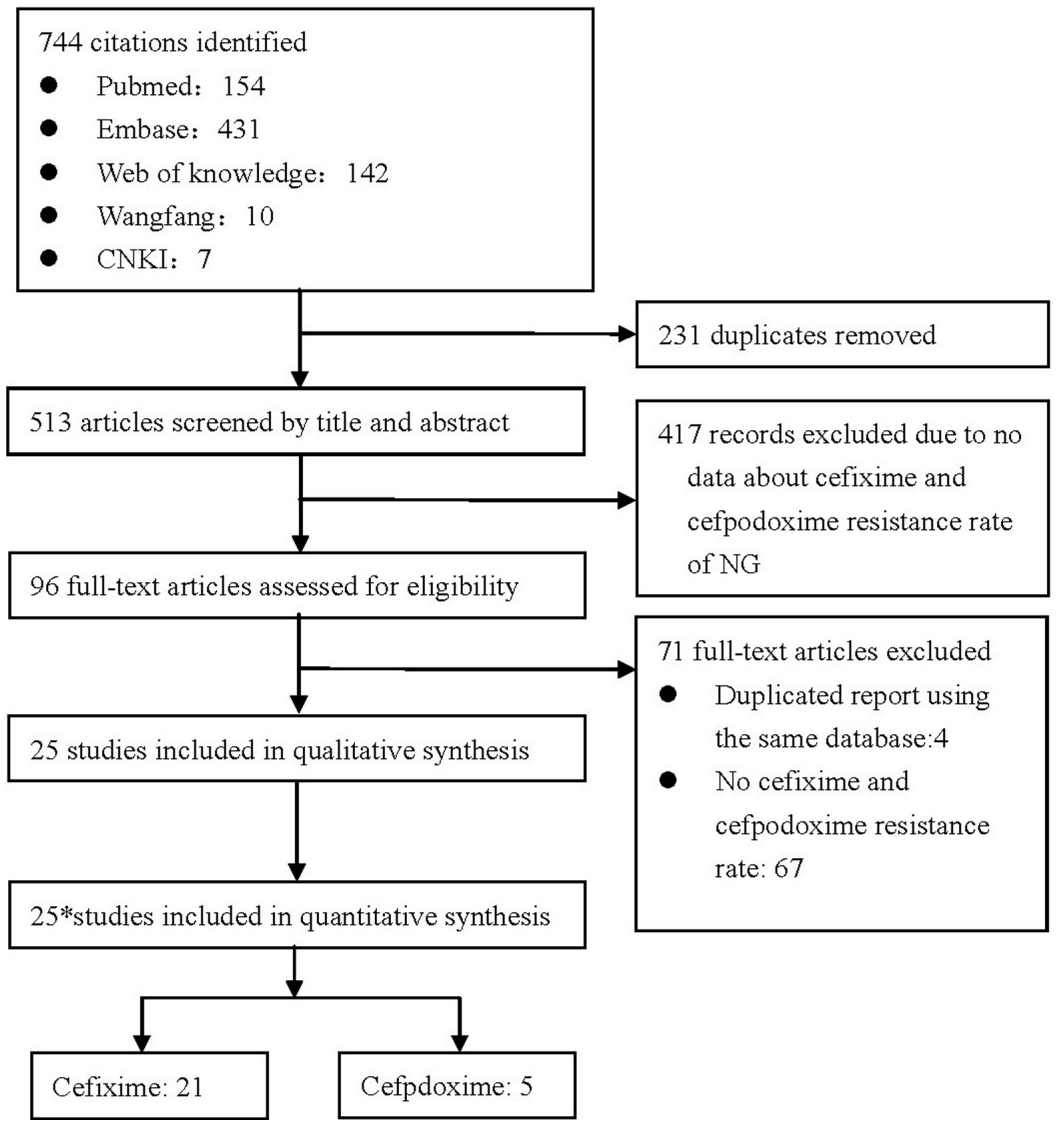
Process of selecting published studies for the meta-analysis according to PRISMA guidelines. * One of the 25 studies contains data concerning. both cefixime and cefpodoxime.

A total of 744 potential abstracts were identified, of which 231 were duplicate records and were thus removed (Figure 1). All of the remaining 513 abstracts were screened, of which 417 were found to have no data concerning cefixime and cefpodoxime susceptibilities and were thus excluded. Therefore, a total of 96 full-text articles were assessed for eligibility; 25 of them were included in the meta-analysis, comprising 21 on antimicrobial susceptibility rate to cefixime and five to cefpodoxime. Of all the included studies, only two studies included the data on susceptibility rates of NG isolates collected after 2010. (Table 1).

### Quality assessment

Among the 25 included studies, only four [27], [28], [29], [30] reported all seven of the categories in our data matrix, seven [31], [32], [33], [34], [35], [36], [37] reported data on six of these categories, ten [38], [39], [40], [41], [42], [43], [44], [45], [46], [47] provided information on five of these categories, and four [48], [49], [50], [51] reported data of only four of these categories (Table 2). The quality of an included study was negatively scored in particular when it failed to describe the study population or NG isolate identification method. Additionally, studies that did not use control strains recommended by the WHO in determining MICs were also scored negatively with respect to quality.

### Meta-analysis

#### Susceptibility rate of NG to cefixime

**Figure 2:**
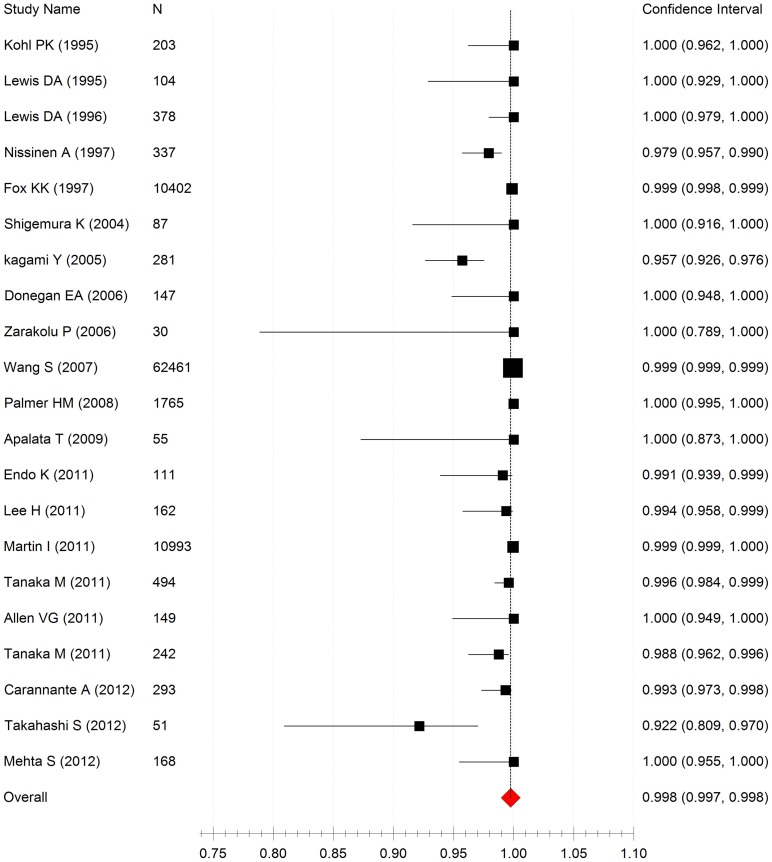
Forest plot of cefixime susceptibility rates in NG isolates from 21 inluded studies. Heterogeneity (I^2^ = 48.1%, P<0.001).

By performing a meta-analysis with fixed model, the pooled susceptibility rate of NG isolates to cefixime was found to be 99.8% (95%CI: 99.7%–99.8%), and evidence of moderate heterogeneity (I^2^ = 48.1%, P<0.001) was observed between included studies (Figure 2). Moreover, significant publication bias was detected (Begg rank correlation test, P = 0.007). In the included 21 studies on cefixime [27], [28], [29], [31], [32], [33], [34], [35], [36], [37], [38], [39], [40], [41], [42], [43], [44], [45], [46], [48], [49], the susceptibility rate of NG isolates to the antibiotic ranged from 92.2% to 100%, with the median of 99.5%. The susceptibility rate was ≥95% in all studies except one conducted in Japan. It reported a susceptibility rate of 92.2% (95% CI: 20.9%–97.0%). Furthermore, non-susceptible isolates were collected from men and the collection period was from January 2007 to January 2009 [45]. In another study conducted in Japan, the susceptibility rate were 99.1% (95%CI: 93.9%–99.9%) after an abnormally low susceptibility rate of 47.4% in 2009 was omitted from the analysis [28].

#### Comparison of cefixime susceptibility rates between included studies

As shown in Table 3, performing a subgroup meta-analysis reduced the heterogeneity of this review. The susceptibility rate of NG isolates from men was lower than that from patients without information on gender or mixed gender group (men and women) (96.5% vs. 99.8%, χ^2^ = 1341.499, P<0.001; 96.5% vs. 99.9%, χ^2^ = 6776.778, P<0.001). There was a statistically significant difference in the cefixime susceptibility rates between different continents (χ^2^ = 692.379, p<0.001); and this rate was lower in Asia than in Europe (97.4% vs. 99.0%, χ^2^ = 669.637, P<0.001) and North America (97.4% vs. 99.9%, χ^2^ = 183.740, P<0.001), or Africa (97.4% vs. 99.5%, χ^2^ = 3.987, P = 0.046). Cefixime susceptibility rate of NG isolates was lower in Japan than in other Asian countries (93.8% vs. 99.3%, χ2 = 6.069, P = 0.014). The NG isolates collected before or during 2003 were more susceptible than those collected after 2003 (99.8% vs. 99.0%, χ2 = 198.597, P<0.001).

**Table 3:** Subgroup analysis for cefixime susceptibility rates.

Subgroup type	Susceptibility rate (95%CI)	No. of the NG isolates	No. of the studies	Heterogeneity		χ^2^ test	
				I^2^ (%)	P-value	χ^2^	P-value
**Population** a							
Men (RS)	96.5 (94.6–97.8)	868	5	0.405	<0.001	-	-
mix	99.9 (99.9–99.9)	76415	7	0.480	<0.001	1341.499	<0.001
Patients	99.8 (99.7–99.9)	11330	6	0.383	0.022	6776.778	<0.001
FSWs	99.7 (94.8–100.0)	147	1	-	-	-	-
Sex workers	98.4 (78.9–99.9)	30	1	-	-	-	-
Young men	99.7 (98.3–99.8)	168	1	-	-	-	-
**Continents**							
Asia (RS)	97.4 (95.7–98.1)	1650	9	0.397	<0.001	-	-
Europe	99.0 (98.1–99.4)	3080	6	0.389	0.017	669.637	<0.001
North America	99.9 (99.9–99.9)	84005	4	0.375	0.054	183.740	<0.001
Africa	99.5 (96.4–99.9)	223	2	<0.001	0.367	3.987	0.046
**Japan or other countries**							
Other Asian countries (RS)	99.3 (97.3–99.8)	339	3	<0.001	0.416	-	-
Japan	93.8 (91.6–95.4)	1311	6	0.468	<0.001	6.069	0.014
Other continents	99.9 (99.8–99.9)	87366	12	0.466	<0.001	3.768	0.052
**Collection period**							
Before or during 2003 (RS)	99.8 (99.8–99.9)	78558	9	0.485	<0.001	-	-
After 2003	99.0 (98.6–99.3)	10202	13	0.460	<0.001	198.597	<0.001
Unknown	99.3 (95.3–99.9)	198	2	<0.001	0.283	-	-
**Overall**	99.8 (99.7–99.8)	88958	21	0.481	<0.001	-	-

a Study population: FSWs (female sex workers); men (men with urethritis); mix (male and female patients), patients (patients whose gender was not identified); young men (young men with discharge); Sex workers (sex workers whose gender was not identified). RS: Reference Subgroup (the subgroups compared with others by χ^2^ test).

#### Distribution of NG isolates with MIC>0.25 mg/L for cefixime

A total of 118 NG isolates with a MIC of >0.25 mg/L for cefixime were identified in 11 studies [28], [29], [32], [35], [37], [39], [40], [43], [45], [49]. The highest number of non-susceptible isolates was found in the United States (n = 60), followed by Japan (n = 42), while substantially lower numbers of such isolates were identified in Finland (n = 7), Canada (n = 6), Italy (n = 2), Korea (n = 1).

#### Susceptibility rate of NG isolates to cefpodoxime

**Figure 3:**
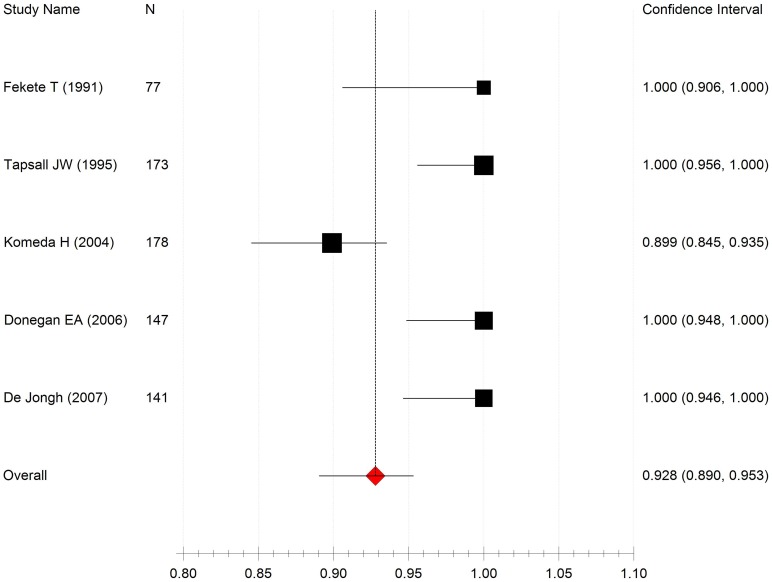
Forest plot of cefpodoxime susceptibility rates in NG isolates from five included studies. Heterogeneity (I^2^ = 44.6%, P<0.001).

Analyzing all the included studies on cefpodoxime susceptibility rates of NG isolates, the pooled susceptibility rate of NG isolates to cefpodoxime was 92.8% (95%CI: 89.0%–95.3%) and evidence for between-study heterogeneity (I^2^ = 44.6%, P<0.001) was observed. No significant publication bias was found (the Begg rank correlation test, P = 0.197). The susceptibility rate of NG isolates to cefpodoxime was lower than that to cefixime (92.8% vs. 99.8%, χ^2^ = 951.809, P<0.001); the former ranged from 89.9% (95% CI: 84.5%–93.5%) to 100% (95% CI: 95.6%–100%) over the five included studies concerning cefpodoxime susceptibility (Figure 3) [27], [30], [47], [50], [51]. Four reported cefpodoxime susceptibility rates of 100%, and one documented a rate of 89.9%. Isolates for this study were collected from men between 1998 and 2002.

## Discussion

Cefixime and cepodoxime are orally administered antimicrobials, with a spectrum of activity against bacterial infections similar to that of ceftriaxone [52]. Although data from previously published studies indicate that NG was generally susceptible to cefixime, evidence from the most current surveillance programs is needed to guide clinical practice. Global and national programs have been developed to improve monitoring of gonococcal resistance, such as the WHO Gonorrhea Antimicrobial Surveillance Programme (GASP) in Asia and Pacific [17], Australian Gonococcal Surveillance Programme (AGSP) in Australia [18], the Gonococcal Resistance to Antimicrobials Surveillance Programme (GRASP) in England and Wales [19] and the Gonococcal Isolate Surveillance Project (GISP) [20] in US. Based on the results from these programs, cefixime has been excluded as a first line drug for treating NG infection in Japan, urban areas of Australia, Europe, and United States. Ongoing use of cefixime may increase the chance of NG developing resistance towards the currently most reliable gonorrhea treatment drug ceftriaxone, which is mediated by the same mosaic Penicillin-Binding Protein 2 (PBP2) [53]. Such increased selective pressure for resistance in gonococci to ceftriaxone may be even more obvious in some specific populations and locations where antibiotics may be widely used (or even misused) in medical practice [54].

In this review, we report that cefixime susceptibility rate of NG isolates from men was lower than that from patients whose gender was not identified, or that from men and women. Consistently, in the past few years, all patients treated unsuccessfully with cefixime were found to be men except one women in one study reported by Allen et al. [6], [7], [8], [9], [10], [15], [16], suggesting that men including men who have sex with men (MSM) are at higher risk of pharyngeal NG infections, which are difficult to treat and where resistant strains are more like to develop [55], [56]. Therefore, it is important to monitor antimicrobial susceptibilities in patients, in particular male patients, to guide gonorrhea antimicrobials treatment, and to better control gonorrhea.

To the best of our knowledge, no prior study has compared cefixime susceptibility rates of NG isolates among different continents. The present study revealed significant differences in susceptibility rate among different continents. Specifically, the cefixime susceptibility rate in Asia was lower than that in other continents, and was particularly low in Japan as compared to other Asian countries. This indicates that NG isolates from Japan were less susceptible to cefixime relative to those from other countries. However, cefixime susceptibility rates in Asia have only been reported in four countries (Japan, Korea, Indonesia, and Turkey), but not in China and India, the two most densely populated countries in the world.

In the included studies, the cefixime susceptibility rate of NG isolates did not appear to significantly change along with the time period (Figure 2). However, the cefixime susceptibility rate in NG isolates collected before or during 2003 was significantly higher than that in isolates collected after 2003, suggesting that worldwide cefixime susceptibility rates in NG isolates has significantly decreased since 2003. Additionally, a decrease in cefixime susceptibility has been noted in certain countries, including Hawaii, Japan, Canada, and Sweden [29], [31], [57], [58]. Although at present NG is still generally susceptible to cefixime worldwide, the susceptibility may decrease with time as the incidence of non-susceptible isolates keeps rising. Furthermore, several studies reported that NG showed a decreased susceptibility to ceftriaxone [31], [45], [59], [60], [61], [62].Thus, it is necessary to develop a wider range of antimicrobial options for super resistant NG strains that are resistant to ESCs as well as other antibiotics [15], [53], [59].

Combining all 21 included studies on cefixime susceptibility, a total of 118 isolates with a MIC of cefixime >0.25 mg/L were identified in six countries, all belonging to developed nations. Among them, the United States had the highest number of non-susceptible NG isolates. Furthermore, the first non-susceptible isolate was also identified there, which might be attributable to the country's advanced and comprehensive monitoring system.

Cefpodoxime has been used as an alternative to cefixime, and the susceptibility rates of NG isolates to the two drugs were previously thought to be similar. However, based on our statistical analysis of the data, the susceptibility rate in NG isolates to cefixime was higher than that to cefpodoxime. The cefpodoxime susceptibility rate reported in Japan was lower than that in other countries where all tested isolates were susceptible to it. Treatment failure with cefpodoxime has not been reported yet, which might be due to the fact that it was less widely used to treat NG infection than cefixime.

To our knowledge, this is the first systematic review of globally published papers on cefixime and cefpodoxime susceptibility rates in NG isolates. Because the included studies varied by quality, the following limitations in this review should be acknowledged. First, some studies had a small sample size of tested NG isolates, resulting in limited statistical power. Second, moderate heterogeneity still existed, which might be attributable to the differences in isolate identification method, media and control strains for MICs testing, and laboratory conditions among included studies. Third, some included studies did not describe the study population and/or isolate collection period. Fourth, there was a selection bias, leading to exclusion of relevant studies from some countries, because MICs of antibiotics were not determined using the standard agar dilution method in those studies. Finally, this review only evaluated published studies, without analyzing original data.

Although in general NG isolates are still highly susceptible to cefixime and cefpodoxime, reduced susceptibility rates were observed in some countries, especially in Japan, and in some populations such as men, which calls for a better control of gonococcal disease and an enhanced global surveillance of drug resistance [3], [63]. Also, given that cefixime susceptibility rate has significantly decreased since 2003, leading to an increasing incidence of treatment failures with cefixime, and emergence of cefixime-resistant NG isolates, more attention should be paid to develop a wider range of antibiotic options and to stress the importance of cautious use of antibiotics. Future studies on the susceptibility rates of NG isolates to cefixime and cefpodoxime should at least take into account the following aspects. First, they need to describe the study population and the demographic characteristics and clinical history of patients, specify the method of strain identification, and use the control strains recommended by the WHO in determining MICs. Second, at least some studies should be conducted in developing countries and those countries, in which use of these antibiotics is not recommended. Third, standard antimicrobial susceptibility testing method should be adopted. In addition, new antibiotic agents should be developed to treat NG infection and their efficacy should be monitored.

## Supporting Information

Table S1 **PubMed Search items.**
(DOC)

Table S2 **PRISMA 2009 checklist of the paper.**
(DOCX)

## References

[1] World Health Organization Department of Reproductive Health and Research. (2011)Prevalence and incidence of selected sexually transmitted infections: Chlamydia trachomatis, Neisseria gonorrhoeae, syphilis and Trichomonasvaginalis. Methods and results used by WHO to generate 2005 estimates 2011. http://www.who.int/ reproductivehealth/publications/rtis/ 9789241502450/en/index.html (19 December 2011, date last accessed)

[2] Barry PM, Klausner JD (2009) The use of cephalosporins for gonorrhea: the impending problem of resistance. Expert Opin Pharmacother 10: 555–577. 19284360 10.1517/14656560902731993 PMC2657229

[3] Deguchi T, Yasuda M, Maeda S (2008) Lack of nationwide surveillance of antimicrobial resistance of Neisseria gonorrhoeae in Japan. Ann Intern Med 149: 363–364. 18765714 10.7326/0003-4819-149-5-200809020-00025

[4] Tapsall JW, Ndowa F, Lewis DA, Unemo M (2009) Meeting the public health challenge of multidrug- and extensively drug-resistant Neisseria gonorrhoeae. Expert Rev Anti Infect Ther 7: 821–834. 19735224 10.1586/eri.09.63

[5] Unemo M, Shafer WM (2011) Antibiotic resistance in Neisseria gonorrhoeae: origin, evolution, and lessons learned for the future. Ann N Y Acad Sci 1230: E19–E28. 22239555 10.1111/j.1749-6632.2011.06215.x PMC4510988

[6] Deguchi T, Yasuda M, Yokoi S, Ishida K, Ito M, et al (2003) Treatment of uncomplicated gonococcal urethritis by double-dosing of 200 mg cefixime at a 6-h interval. J Infect Chemother 9: 35–39. 12673405 10.1007/s10156-002-0204-8

[7] Yokoi S, Deguchi T, Ozawa T, Yasuda M, Ito S, et al (2007) Threat to cefixime treatment for gonorrhea. Emerg Infect Dis 13: 1275–1277. 17953118 10.3201/eid1308.060948 PMC2828067

[8] Unemo M, Golparian D, Syversen G, Vestrheim DF, Moi H (2010) Two cases of verified clinical failures using internationally recommended first-line cefixime for gonorrhoea treatment, Norway, 2010. Euro Surveill 15. 10.2807/ese.15.47.19721-en 21144442

[9] Unemo M, Golparian D, Stary A, Eigentler A (2011) First Neisseria gonorrhoeae strain with resistance to cefixime causing gonorrhoea treatment failure in Austria, 2011. Euro Surveill 16. 22085601

[10] Ison CA, Hussey J, Sankar KN, Evans J, Alexander S (2011) Gonorrhoea treatment failures to cefixime and azithromycin in England, 2010. Euro Surveill 16. 21492528

[11] Tapsall J, Read P, Carmody C, Bourne C, Ray S, et al (2009) Two cases of failed ceftriaxone treatment in pharyngeal gonorrhoea verified by molecular microbiological methods. J Med Microbiol 58: 683–687. 19369534 10.1099/jmm.0.007641-0

[12] Unemo M, Golparian D, Hestner A (2011) Ceftriaxone treatment failure of pharyngeal gonorrhoea verified by international recommendations, Sweden, July 2010. Euro Surveill 16. 21329645

[13] Lo JY, Ho KM, Leung AO, Tiu FS, Tsang GK, et al (2008) Ceftibuten resistance and treatment failure of Neisseria gonorrhoeae infection. Antimicrob Agents Chemother 52: 3564–3567. 18663018 10.1128/AAC.00198-08 PMC2565891

[14] Akasaka S, Muratani T, Yamada Y, Inatomi H, Takahashi K, et al (2001) Emergence of cephem- and aztreonam-high-resistant Neisseria gonorrhoeae that does not produce beta-lactamase. J Infect Chemother 7: 49–50. 11406757 10.1007/s101560170034

[15] Unemo M, Golparian D, Nicholas R, Ohnishi M, Gallay A, et al (2012) High-level cefixime- and ceftriaxone-resistant Neisseria gonorrhoeae in France: novel penA mosaic allele in a successful international clone causes treatment failure. Antimicrob Agents Chemother 56: 1273–1280. 22155830 10.1128/AAC.05760-11 PMC3294892

[16] Allen VG, Mitterni L, Seah C, Rebbapragada A, Martin IE, et al (2013) Neisseria gonorrhoeae treatment failure and susceptibility to cefixime in Toronto, Canada. JAMA 309: 163–170. 23299608 10.1001/jama.2012.176575

[17] Australian Gonococcal Surveillance Programme (2011) Australian Gonococcal Surveillance Programme annual report, 2010. Commun Dis Intell Q Rep 35: 229–236. 22624483 10.33321/cdi.2011.35.21

[18] Tapsall JW (2009) Implications of current recommendations for third-generation cephalosporin use in the WHO Western Pacific Region following the emergence of multiresistant gonococci. Sex Transm Infect 85: 256–258. 19261600 10.1136/sti.2008.035337

[19] Unemo M (2012) The 2012 European guideline on the diagnosis and treatment of gonorrhoea in adults recommends dual antimicrobial therapy. Euro Surveill 17. 10.2807/ese.17.47.20323-en 23231859

[20] Maldonado NG, Takhar SS (2013) Update on Emerging Infections: news from the Centers for Disease Control and Prevention. Update to the CDC's Sexually Transmitted Diseases Treatment Guidelines, 2010: Oral cephalosporins no longer a recommended treatment for gonococcal infections. Ann Emerg Med 61: 91–95. 23260686 10.1016/j.annemergmed.2012.10.015

[21] Kropp RY, Latham-Carmanico C, Steben M, Wong T, Duarte-Franco E (2007) What's new in management of sexually transmitted infections? Canadian Guidelines on Sexually Transmitted Infections, 2006 Edition. Can Fam Physician 53: 1739–1741. 17934039 PMC2231441

[22] Institutes CALS (2008) Performance Standards for Antimicrobial Susceptibility Testing: Eighteenth Informational Supplement M100-S18.

[23] Moher D, Liberati A, Tetzlaff J, Altman DG (2009) Preferred reporting items for systematic reviews and meta-analyses: the PRISMA statement. PLoS Med 6: e1000097. 19621072 10.1371/journal.pmed.1000097 PMC2707599

[24] Liberati A, Altman DG, Tetzlaff J, Mulrow C, Gotzsche PC, et al (2009) The PRISMA statement for reporting systematic reviews and meta-analyses of studies that evaluate health care interventions: explanation and elaboration. PLoS Med 6: e1000100. 19621070 10.1371/journal.pmed.1000100 PMC2707010

[25] Higgins JP, Thompson SG, Deeks JJ, Altman DG (2003) Measuring inconsistency in meta-analyses. BMJ 327: 557–560. 12958120 10.1136/bmj.327.7414.557 PMC192859

[26] Borenstein M, Hedges LV, Higgins JP, Rothstein HR (2011) Introduction to meta-analysis: Wiley press, 277–291p

[27] Donegan EA, Wirawan DN, Muliawan P, Schachter J, Moncada J, et al (2006) Fluoroquinolone-resistant Neisseria gonorrhoeae in Bali, Indonesia: 2004. Sex Transm Dis 33: 625–629. 16601661 10.1097/01.olq.0000216012.83990.bd

[28] Endo K, Onodera S, Kiyota H, Suzuki H, Hosobe T, et al (2011) Drug-susceptibilities of Neisseria gonorrhoeae strains isolated from male patients with gonococcal urethritis against antimicrobial agents - Comparisons from 2006 to 2010. Japanese Journal of Chemotherapy 59: 308–312.

[29] Tanaka M, Koga Y, Nakayama H, Kanayama A, Kobayashi I, et al (2011) Antibiotic-resistant phenotypes and genotypes of Neisseria gonorrhoeae isolates in Japan: identification of strain clusters with multidrug-resistant phenotypes. Sex Transm Dis 38: 871–875. 21844744 10.1097/OLQ.0b013e31821d0f98

[30] De Jongh M, Dangor Y, Adam A, Hoosen AA (2007) Gonococcal resistance: evolving from penicillin, tetracycline to the quinolones in South Africa – implications for treatment guidelines. Int J STD AIDS 18: 697–699. 17945048 10.1258/095646207782193768

[31] Allen VG, Farrell DJ, Rebbapragada A, Tan J, Tijet N, et al (2011) Molecular analysis of antimicrobial resistance mechanisms in Neisseria gonorrhoeae isolates from Ontario, Canada. Antimicrob Agents Chemother 55: 703–712. 21098249 10.1128/AAC.00788-10 PMC3028768

[32] Carannante A, Prignano G, Cusini M, Matteelli A, Dal Conte I, et al (2012) Cefixime and ceftriaxone susceptibility of Neisseria gonorrhoeae in Italy from 2006 to 2010. Clin Microbiol Infect 18: 558–564. 21966997 10.1111/j.1469-0691.2011.03619.x

[33] Lee H, Hong SG, Soe Y, Yong D, Jeong SH, et al (2011) Trends in antimicrobial resistance of Neisseria gonorrhoeae isolated from Korean patients from 2000 to 2006. Sex Transm Dis 38: 1082–1086. 21992988 10.1097/OLQ.0b013e31822e60a4

[34] Lewis DA, Ison CA, Forster GE, Goh BT (1996) Tetracycline-resistant Neisseria gonorrhoeae. Characteristics of patients and isolates at a London Genitourinary Medicine Clinic. Sex Transm Dis 23: 378–383. 8885068 10.1097/00007435-199609000-00006

[35] Nissinen A, Jarvinen H, Liimatainen O, Jahkola M, Huovinen P (1997) Antimicrobial resistance in Neisseria gonorrhoeae in Finland, 1976 to 1995. The Finnish Study Group for Antimicrobial Resistance. Sex Transm Dis 24: 576–581. 9383846 10.1097/00007435-199711000-00005

[36] Mehta S, Maclean I, ndinya-Acholam J, Murugu R, Agunda L, et al (2012) A RonaldAntimicrobial resistance to neisseria gonorrhoeae in a cohort young men in Kisumu, Kenya 2000–2009. Sex Transm infect 87: A26.

[37] Wang SA, Harvey AB, Conner SM, Zaidi AA, Knapp JS, et al (2007) Antimicrobial resistance for Neisseria gonorrhoeae in the United States, 1988 to 2003: the spread of fluoroquinolone resistance. Ann Intern Med 147: 81–88. 17638718 10.7326/0003-4819-147-2-200707170-00006

[38] Apalata T, Zimba TF, Sturm WA, Moodley P (2009) Antimicrobial susceptibility profile of Neisseria gonorrhoeae isolated from patients attending a STD facility in Maputo, Mozambique. Sex Transm Dis 36: 341–343. 19556927 10.1097/OLQ.0b013e3181982e3c

[39] Fox KK, Knapp JS, Holmes KK, Hook ER, Judson FN, et al (1997) Antimicrobial resistance in Neisseria gonorrhoeae in the United States, 1988–1994: the emergence of decreased susceptibility to the fluoroquinolones. J Infect Dis 175: 1396–1403. 9180179 10.1086/516472

[40] Kagami Y, Endo K, Suzuki H, Kiyota H, Onodera S (2005) Drug-susceptibilities of Neisseria gonorrhoeae strains isolated from male patients with gonococcal urethritis against antimicrobial agents-Their comparisons from 1999 to 2004. Japanese Journal of Chemotherapy 53: 483–487.

[41] Kohl PK, Tu Y, Hostalek U, Petzoldt D (1995) Activity of cefixime against *Neisseria gonorrhoeae* . Journal of the European Academy of Dermatology and Venereology 4: 155–159.

[42] Lewis DA, Ison CA, Livermore DM, Chen HY, Hooi AY, et al (1995) A one-year survey of Neisseria gonorrhoeae isolated from patients attending an east London genitourinary medicine clinic: antibiotic susceptibility patterns and patients' characteristics. Genitourin Med 71: 13–17. 7750947 10.1136/sti.71.1.13 PMC1195362

[43] Martin I, Jayaraman G, Wong T, Liu G, Gilmour M (2011) Trends in antimicrobial resistance in Neisseria gonorrhoeae isolated in Canada: 2000–2009. Sex Transm Dis 38: 892–898. 21934558 10.1097/OLQ.0b013e31822c664f

[44] Shigemura K, Okada H, Shirakawa T, Tanaka K, Arakawa S, et al (2004) Susceptibilities of Neisseria gonorrhoeae to fluoroquinolones and other antimicrobial agents in Hyogo and Osaka, Japan. Sex Transm Infect 80: 105–107. 15054169 10.1136/sti.2003.006908 PMC1744816

[45] Takahashi S, Kurimura Y, Hashimoto J, Uehara T, Hiyama Y, et al (2013) Antimicrobial susceptibility and penicillin-binding protein 1 and 2 mutations in Neisseria gonorrhoeae isolated from male urethritis in Sapporo, Japan. J Infect Chemother 19: 50–56. 22797875 10.1007/s10156-012-0450-3

[46] Zarakolu P, Sakizligil B, Unal S (2006) Antimicrobial resistance of Neisseria gonorrhoeae strains isolated from sex workers in Ankara]. Mikrobiyol Bul 40: 69. 16775959

[47] Komeda H, Fujimoto Y, Uno M, Asano Y, Isogai K (2004) Surveillance of susceptibility to antimicrobial agents of Neisseria gonorrhoeae isolated from male urethritis. Japanese Journal of Chemotherapy 52: 31–34.

[48] Palmer HM, Young H, Graham C, Dave J (2008) Prediction of antibiotic resistance using Neisseria gonorrhoeae multi-antigen sequence typing. Sex Transm Infect 84: 280–284. 18256103 10.1136/sti.2008.029694

[49] Tanaka M, Shimojima M, Saika T, Iyoda T, Ikeda F, et al (2011) [Nationwide antimicrobial susceptibility survey of Neisseria gonorrhoeae isolates in Japan]. Kansenshogaku Zasshi 85: 360–365. 21861439 10.11150/kansenshogakuzasshi.85.360

[50] Fekete T, Woodwell J, Cundy KR (1991) Susceptibility of Neisseria gonorrhoeae to cefpodoxime: determination of MICs and disk diffusion zone diameters. Antimicrob Agents Chemother 35: 497–499. 1903910 10.1128/aac.35.3.497 PMC245038

[51] Tapsall JW, Phillips EA (1995) The sensitivity of 173 Sydney isolates of Neisseria gonorrhoeae to cefpodoxime and other antibiotics used to treat gonorrhea. Pathology 27: 64–66. 7603756 10.1080/00313029500169492

[52] Plourde PJ, Tyndall M, Agoki E, Ombette J, Slaney LA, et al (1992) Single-dose cefixime versus single-dose ceftriaxone in the treatment of antimicrobial-resistant Neisseria gonorrhoeae infection. J Infect Dis 166: 919–922. 1527431 10.1093/infdis/166.4.919

[53] Camara J, Serra J, Ayats J, Bastida T, Carnicer-Pont D, et al (2012) Molecular characterization of two high-level ceftriaxone-resistant Neisseria gonorrhoeae isolates detected in Catalonia, Spain. J Antimicrob Chemother 67: 1858–1860. 22566592 10.1093/jac/dks162

[54] Kotwani A, Wattal C, Joshi PC, Holloway K (2012) Irrational use of antibiotics and role of the pharmacist: an insight from a qualitative study in New Delhi, India. J Clin Pharm Ther 37: 308–312. 21883328 10.1111/j.1365-2710.2011.01293.x

[55] Lewis DA, Sriruttan C, Muller EE, Golparian D, Gumede L, et al (2013) Phenotypic and genetic characterization of the first two cases of extended-spectrum-cephalosporin-resistant Neisseria gonorrhoeae infection in South Africa and association with cefixime treatment failure. J Antimicrob Chemother 68: 1267–1270. 23416957 10.1093/jac/dkt034

[56] Kirkcaldy RD, Zaidi A, Hook ER, Holmes KH, Soge O, et al (2013) Neisseria gonorrhoeae antimicrobial resistance among men who have sex with men and men who have sex exclusively with women: the Gonococcal Isolate Surveillance Project, 2005–2010. Ann Intern Med 158: 321–328. 23460055 10.7326/0003-4819-158-5-201303050-00004 PMC6697257

[57] Wang SA, Lee MV, O'Connor N, Iverson CJ, Ohye RG, et al (2003) Multidrug-resistant Neisseria gonorrhoeae with decreased susceptibility to cefixime-Hawaii, 2001. Clin Infect Dis 37: 849–852. 12955650 10.1086/377500

[58] Lindberg R, Fredlund H, Nicholas R, Unemo M (2007) Neisseria gonorrhoeae isolates with reduced susceptibility to cefixime and ceftriaxone: association with genetic polymorphisms in penA, mtrR, porB1b, and ponA. Antimicrob Agents Chemother 51: 2117–2122. 17420216 10.1128/AAC.01604-06 PMC1891421

[59] Goire N, Ohnishi M, Limnios AE, Lahra MM, Lambert SB, et al (2012) Enhanced gonococcal antimicrobial surveillance in the era of ceftriaxone resistance: a real-time PCR assay for direct detection of the Neisseria gonorrhoeae H041 strain. J Antimicrob Chemother 67: 902–905. 22207596 10.1093/jac/dkr549

[60] Su X, Jiang F, Qimuge, Dai X, Sun H, et al (2007) Surveillance of antimicrobial susceptibilities in Neisseria gonorrhoeae in Nanjing, China, 1999–2006. Sex Transm Dis 34: 995–999. 17595594 10.1097/OLQ.0b013e3180ca8f24

[61] Ito M, Yasuda M, Yokoi S, Ito S, Takahashi Y, et al (2004) Remarkable increase in central Japan in 2001–2002 of Neisseria gonorrhoeae isolates with decreased susceptibility to penicillin, tetracycline, oral cephalosporins, and fluoroquinolones. Antimicrob Agents Chemother 48: 3185–3187. 15273147 10.1128/AAC.48.8.3185-3187.2004 PMC478532

[62] Annual Report of the Australian Gonococcal Surveillance Programme, 2009. Commun Dis Intell Q Rep 34: 89–95. 10.33321/cdi.2010.34.12 20690208

[63] Workowski KA, Berman SM, Douglas JJ (2008) Emerging antimicrobial resistance in Neisseria gonorrhoeae: urgent need to strengthen prevention strategies. Ann Intern Med 148: 606–613. 18413622 10.7326/0003-4819-148-8-200804150-00005

