# Supplementary material for: Worldwide Susceptibility Rates of Neisseria gonorrhoeae Isolates to Cefixime and Cefpodoxime: A Systematic Review and Meta-Analysis
Source: PLoS One. 2014 Jan 31;9(1):e87849. doi: 10.1371/journal.pone.0087849 (PMC3909252; doi:10.1371/journal.pone.0087849)
Supplement: Table S1 — PubMed Search items. (DOC) [file pone.0087849.s001.doc]

**Table S1. PubMed Search items**

| Search | Add to builder | Query | Items found | Time (15 Oct. 2012) |
| --- | --- | --- | --- | --- |
| #1 | Add | Search((neisseria gonorrhoeae) OR gonococcus) OR gonorrhea | 20222 | 22:06:15 |
| #2 | Add | Search (cefixime) OR cefpodoxime | 1718 | 22:07:28 |
| #3 | Add | Search ((((((resistance) OR susceptibility) OR resistant) OR susceptible) OR MIC) OR minimum inhibitory concentration) OR MICs | 938672 | 22:10:18 |
| #4 | Add | Search ((#1 AND #2) AND #3 | 146 | 22:10:46 |
| #5 | Add | Search “Neisseria gonorrhoeae” [Mesh] | 7932 | 22:12:52 |
| #6 | Add | Search “cefixime” [Mesh] | 594 | 22:13:30 |
| #7 | Add | Search “cefpodoxime” [supplementary concept] | 162 | 22:14:24 |
| #8 | Add | Search ((#6) OR #7) AND #5 | 53 | 22:15:25 |
| #9 | Add | Search (#5) AND #2 | 108 | 22:17:46 |
| #10 | Add | Search ((#4) OR #8) OR #9 | 154 | 22:33:51 |
